# 9α-Bromo analog of beclometasone dipropionate monohydrate

**DOI:** 10.1107/S1600536809025975

**Published:** 2009-07-11

**Authors:** Kamal Aziz Ketuly, A. Hamid A. Hadi, Seik Weng Ng

**Affiliations:** a Department of Chemistry, University of Malaya, 50603 Kuala Lumpur, Malaysia

## Abstract

In the crystal structure of (8*S*,9*R*,10*S*,11*S*,13*S*,14*S*,16*S*,17*R*)-9α-bromo-11-hydr­oxy-10,13,16-trimethyl-3-oxo-17-[2-(propion­yloxy)acet­yl]-6,7,8,9,10,11,12,13,14,15,16,17-dodeca­hydro-3*H*-cyclo­penta­[*a*]phenanthren-17-yl propionate monohydrate, C_28_H_37_BrO_7_·H_2_O, which has a 9α-Br atom in place of the 9α-Cl atom of monohydrated beclometasone dipropionate, one six-membered ring is planar (r.m.s. deviation = 0.02 Å) owing to its 1,4-diene-3-one composition, whereas the two other six-membered rings each have a chair conformation. The organic mol­ecule and water mol­ecules engage in hydrogen-bonding inter­actions, generating a helical chain running along the *c* axis of the ortho­rhom­bic unit cell.

## Related literature

For the NMR data and the crystal structure of the asthma drug beclometasone dipropionate monohydrate, see: Othman *et al.* (2008 ▶); Duax *et al.* (1981 ▶). The two compounds are isostructural.
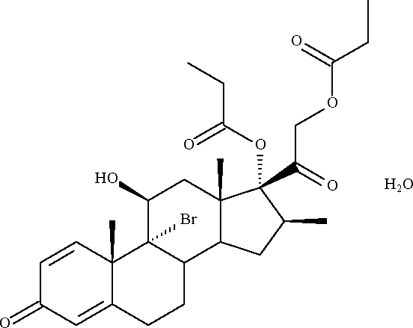


## Experimental

### 

#### Crystal data

C_28_H_37_BrO_7_·H_2_O
*M* *_r_* = 583.50
Orthorhombic, 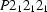

*a* = 11.9565 (2) Å
*b* = 14.1329 (2) Å
*c* = 16.1648 (3) Å
*V* = 2731.53 (8) Å^3^
*Z* = 4
Mo *K*α radiation
μ = 1.55 mm^−1^
*T* = 140 K
0.40 × 0.08 × 0.08 mm

#### Data collection

Bruker SMART APEX diffractometer
Absorption correction: multi-scan (*SADABS*; Sheldrick, 1996 ▶) *T*_min_ = 0.611, *T*_max_ = 0.746 (expected range = 0.723–0.883)
18917 measured reflections
6226 independent reflections
5575 reflections with *I* > 2σ(*I*)
*R*_int_ = 0.028

#### Refinement

*R*[*F* ^2^ > 2σ(*F* ^2^)] = 0.026
*wR*(*F* ^2^) = 0.055
*S* = 0.96
6226 reflections
351 parameters
4 restraints
H atoms treated by a mixture of independent and constrained refinement
Δρ_max_ = 0.39 e Å^−3^
Δρ_min_ = −0.25 e Å^−3^
Absolute structure: Flack (1983 ▶), 2717 Friedel pairs
Flack parameter: 0.001 (5)

### 

Data collection: *APEX2* (Bruker, 2008 ▶); cell refinement: *SAINT* (Bruker, 2008 ▶); data reduction: *SAINT*; program(s) used to solve structure: *SHELXS97* (Sheldrick, 2008 ▶); program(s) used to refine structure: *SHELXL97* (Sheldrick, 2008 ▶); molecular graphics: *X-SEED* (Barbour, 2001 ▶); software used to prepare material for publication: *publCIF* (Westrip, 2009 ▶).

## Supplementary Material

Crystal structure: contains datablocks global, I. DOI: 10.1107/S1600536809025975/tk2488sup1.cif

Structure factors: contains datablocks I. DOI: 10.1107/S1600536809025975/tk2488Isup2.hkl

Additional supplementary materials:  crystallographic information; 3D view; checkCIF report

## Figures and Tables

**Table 1:** Hydrogen-bond geometry (Å, °)

*D*—H⋯*A*	*D*—H	H⋯*A*	*D*⋯*A*	*D*—H⋯*A*
O2—H2⋯O1w	0.83 (1)	1.93 (1)	2.759 (2)	173 (2)
O1w—H11⋯O5	0.84 (1)	2.06 (1)	2.859 (2)	159 (3)
O1w—H12⋯O7^i^	0.84 (1)	2.03 (1)	2.854 (2)	168 (2)

Symmetry code: (i) 
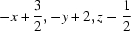
.

**Fig. 1.**
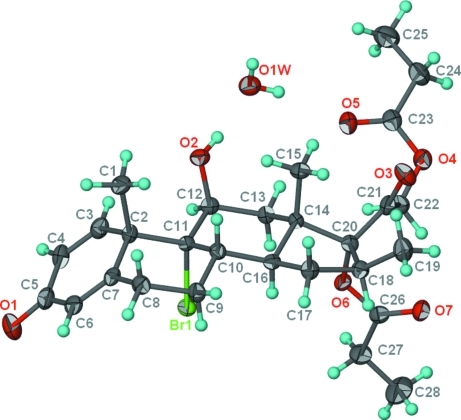
Thermal ellipsoid plot (Barbour, 2001) of C28H37BrO7.H2O at the 70% probability level. Hydrogen atoms are drawn as spheres of arbitrary radius.

## supplementary crystallographic information

### Experimental

17α,21-Dihydroxy-16β-methylpregna-1,4,9-triene-3,20-dionedipropionate (5.5 g) was reacted with bromodan (1,3-dibromo-5,5-dimethylhydantion) (4.8 g) to give a yellow product (6.6 g). TLC showed one major product and five minor ones. The major product (3.0 g) was initially recrystallized three times, once from chloroform-methanol (1:2 v/v), and the second and third times from hexane-methanol (1:1 v/v). The recovered material (1.8 g). was then divided for two different recrystallizations.

Recrystallization form ethyl acetate-methanol (1:9 v/v) gave a white powder, m.p. 453–455 K. Elemental analysis: Calc. for C_28_H_37_BrO_7_ (mol. wt. 564): C 59.47, H 6.59, Br 14.13%. Found: C 59.3, H 6.57, Br 13.83%.

Recrystallization from methanol water gave colorless crystals, m.p. 460–461 K. Elemental analysis: Calc. for C_22_H_37_BrO_7_^.^H_2_O (mol. wt. 582): C 57.64, H 6.69, Br 13.70%. Found: C 57.68, H 6.78, Br 13.46%. The water molecule was detected from the ^1^H-NMR spectrum. Thermogravimetric analysis showed a peak at 365 K that corresponded to the loss of water.

### Refinement

Hydrogen atoms were placed at calculated positions (C–H 0.95–1.00 Å) and were treated as riding on their parent carbon atoms, with *U*(H) set to 1.2–1.5 times *U*_eq_(*C*). The O-H atoms were refined with 0.84 (1) Å and with *U*(H) set to 1.5 times *U*_eq_(*O*).

### Crystal data

**Table:**

C_28_H_37_BrO_7_·H_2_O	*F*(000) = 1224
*M**_r_* = 583.50	*D*_x_ = 1.419 Mg m^−^^3^
Orthorhombic, *P*2_1_2_1_2_1_	Mo *K*α radiation, λ = 0.71073 Å
Hall symbol: P 2ac 2ab	Cell parameters from 7177 reflections
*a* = 11.9565 (2) Å	θ = 2.2–28.2°
*b* = 14.1329 (2) Å	µ = 1.55 mm^−^^1^
*c* = 16.1648 (3) Å	*T* = 140 K
*V* = 2731.53 (8) Å^3^	Prism, brown
*Z* = 4	0.40 × 0.08 × 0.08 mm

### Data collection

**Table:**

Bruker SMART APEX diffractometer	6226 independent reflections
Radiation source: fine-focus sealed tube	5575 reflections with *I* > 2σ(*I*)
graphite	*R*_int_ = 0.028
ω scans	θ_max_ = 27.5°, θ_min_ = 1.9°
Absorption correction: multi-scan (*SADABS*; Sheldrick, 1996)	*h* = −15→15
*T*_min_ = 0.611, *T*_max_ = 0.746	*k* = −18→18
18917 measured reflections	*l* = −20→21

### Refinement

**Table:**

Refinement on *F*^2^	Secondary atom site location: difference Fourier map
Least-squares matrix: full	Hydrogen site location: inferred from neighbouring sites
*R*[*F*^2^ > 2σ(*F*^2^)] = 0.026	H atoms treated by a mixture of independent and constrained refinement
*wR*(*F*^2^) = 0.055	*w* = 1/[σ^2^(*F*_o_^2^) + (0.0102*P*)^2^] where *P* = (*F*_o_^2^ + 2*F*_c_^2^)/3
*S* = 0.96	(Δ/σ)_max_ = 0.001
6226 reflections	Δρ_max_ = 0.39 e Å^−^^3^
351 parameters	Δρ_min_ = −0.25 e Å^−^^3^
4 restraints	Absolute structure: Flack (1983), 2717 Friedel pairs
Primary atom site location: structure-invariant direct methods	Flack parameter: 0.001 (5)

### Fractional atomic coordinates and isotropic or equivalent isotropic displacement parameters (Å^2^)

**Table:**

	*x*	*y*	*z*	*U*_iso_*/*U*_eq_	
Br1	0.657482 (17)	0.615717 (12)	0.957131 (12)	0.01918 (5)	
O1	0.66966 (16)	0.34385 (10)	0.78558 (10)	0.0357 (4)	
O2	0.81449 (12)	0.80839 (9)	0.80872 (9)	0.0228 (3)	
H2	0.8635 (13)	0.8476 (11)	0.8218 (12)	0.018 (6)*	
O3	0.67741 (12)	1.10469 (9)	1.01228 (9)	0.0272 (3)	
O4	0.86491 (12)	1.09748 (9)	1.10396 (9)	0.0247 (3)	
O5	0.93019 (13)	1.02972 (10)	0.98878 (9)	0.0275 (4)	
O6	0.64117 (12)	0.87198 (8)	1.09415 (7)	0.0177 (3)	
O7	0.56588 (13)	0.95956 (9)	1.19554 (9)	0.0233 (3)	
O1W	0.98752 (14)	0.93262 (10)	0.83951 (10)	0.0274 (3)	
H11	0.989 (2)	0.9626 (15)	0.8845 (8)	0.042 (8)*	
H12	0.975 (3)	0.9715 (14)	0.8012 (10)	0.061 (10)*	
C1	0.6947 (2)	0.69388 (14)	0.69871 (12)	0.0247 (5)	
H1A	0.6844	0.6503	0.6521	0.037*	
H1B	0.7695	0.7222	0.6957	0.037*	
H1C	0.6381	0.7439	0.6961	0.037*	
C2	0.68233 (17)	0.63823 (12)	0.78206 (12)	0.0191 (4)	
C3	0.78273 (19)	0.57607 (15)	0.78750 (13)	0.0225 (5)	
H3	0.8537	0.6058	0.7923	0.027*	
C4	0.7804 (2)	0.48178 (14)	0.78608 (13)	0.0249 (5)	
H4	0.8487	0.4474	0.7873	0.030*	
C5	0.6745 (2)	0.43061 (14)	0.78268 (13)	0.0250 (5)	
C6	0.57302 (19)	0.48835 (14)	0.77512 (12)	0.0222 (5)	
H6	0.5031	0.4570	0.7696	0.027*	
C7	0.57411 (18)	0.58267 (14)	0.77566 (12)	0.0195 (4)	
C8	0.46949 (17)	0.64072 (13)	0.77038 (13)	0.0207 (4)	
H8A	0.4036	0.5983	0.7693	0.025*	
H8B	0.4700	0.6779	0.7185	0.025*	
C9	0.46048 (17)	0.70818 (13)	0.84465 (13)	0.0198 (4)	
H9A	0.3963	0.7514	0.8362	0.024*	
H9B	0.4460	0.6709	0.8954	0.024*	
C10	0.56683 (17)	0.76670 (12)	0.85667 (12)	0.0165 (4)	
H10	0.5743	0.8098	0.8080	0.020*	
C11	0.67279 (18)	0.70477 (12)	0.86053 (11)	0.0159 (4)	
C12	0.78016 (17)	0.76116 (13)	0.88200 (12)	0.0178 (4)	
H12A	0.8397	0.7146	0.8970	0.021*	
C13	0.76505 (16)	0.82939 (12)	0.95594 (13)	0.0162 (4)	
H13A	0.7597	0.7919	1.0075	0.019*	
H13B	0.8319	0.8704	0.9603	0.019*	
C14	0.66039 (16)	0.89215 (11)	0.94808 (11)	0.0156 (3)	
C15	0.67492 (18)	0.96648 (13)	0.87915 (11)	0.0197 (4)	
H15A	0.6775	0.9347	0.8253	0.030*	
H15B	0.7448	1.0013	0.8880	0.030*	
H15C	0.6118	1.0107	0.8803	0.030*	
C16	0.55793 (17)	0.82829 (13)	0.93416 (12)	0.0158 (4)	
H16	0.5538	0.7843	0.9825	0.019*	
C17	0.46005 (16)	0.89684 (13)	0.94236 (12)	0.0196 (4)	
H17A	0.3890	0.8625	0.9518	0.023*	
H17B	0.4525	0.9367	0.8923	0.023*	
C18	0.49199 (17)	0.95706 (13)	1.01841 (12)	0.0188 (4)	
H18	0.4556	0.9265	1.0673	0.023*	
C19	0.44583 (19)	1.05760 (13)	1.01391 (14)	0.0266 (5)	
H19A	0.4666	1.0922	1.0642	0.040*	
H19B	0.3642	1.0553	1.0092	0.040*	
H19C	0.4771	1.0899	0.9655	0.040*	
C20	0.62151 (17)	0.94357 (12)	1.02997 (12)	0.0172 (4)	
C21	0.69204 (16)	1.03130 (12)	1.04845 (13)	0.0194 (4)	
C22	0.78712 (19)	1.02089 (14)	1.11030 (13)	0.0237 (5)	
H22A	0.7560	1.0190	1.1670	0.028*	
H22B	0.8266	0.9604	1.1002	0.028*	
C23	0.93404 (17)	1.09329 (12)	1.03848 (14)	0.0223 (4)	
C24	1.01341 (19)	1.17575 (13)	1.03599 (16)	0.0292 (5)	
H24A	1.0313	1.1949	1.0934	0.035*	
H24B	0.9758	1.2298	1.0087	0.035*	
C25	1.1207 (2)	1.15487 (16)	0.99096 (16)	0.0361 (6)	
H25A	1.1657	1.2126	0.9875	0.054*	
H25B	1.1036	1.1322	0.9351	0.054*	
H25C	1.1625	1.1061	1.0210	0.054*	
C26	0.60442 (16)	0.88507 (15)	1.17195 (11)	0.0194 (4)	
C27	0.6195 (2)	0.79691 (15)	1.22196 (13)	0.0289 (5)	
H27A	0.5740	0.7459	1.1968	0.035*	
H27B	0.6989	0.7774	1.2186	0.035*	
C28	0.5879 (3)	0.80587 (18)	1.31099 (15)	0.0490 (8)	
H28A	0.6052	0.7467	1.3399	0.074*	
H28B	0.5077	0.8190	1.3154	0.074*	
H28C	0.6303	0.8578	1.3362	0.074*	

### Atomic displacement parameters (Å^2^)

**Table:**

	*U*^11^	*U*^22^	*U*^33^	*U*^12^	*U*^13^	*U*^23^
Br1	0.02274 (10)	0.01686 (8)	0.01795 (8)	0.00030 (8)	0.00010 (9)	0.00231 (8)
O1	0.0466 (12)	0.0182 (7)	0.0421 (10)	0.0026 (8)	0.0022 (9)	−0.0055 (7)
O2	0.0226 (9)	0.0247 (7)	0.0211 (7)	−0.0069 (6)	0.0043 (7)	−0.0014 (6)
O3	0.0291 (9)	0.0165 (6)	0.0361 (8)	0.0000 (6)	−0.0037 (7)	0.0020 (6)
O4	0.0224 (9)	0.0257 (7)	0.0261 (7)	−0.0047 (6)	0.0011 (6)	−0.0071 (6)
O5	0.0310 (9)	0.0239 (7)	0.0276 (8)	−0.0022 (7)	0.0026 (7)	−0.0074 (6)
O6	0.0216 (8)	0.0173 (6)	0.0143 (6)	0.0028 (6)	0.0019 (6)	0.0018 (5)
O7	0.0281 (9)	0.0220 (7)	0.0197 (7)	0.0036 (6)	0.0027 (7)	−0.0024 (6)
O1W	0.0315 (9)	0.0279 (8)	0.0227 (8)	−0.0049 (7)	0.0016 (8)	0.0012 (7)
C1	0.0295 (13)	0.0280 (10)	0.0167 (10)	−0.0050 (9)	0.0023 (9)	−0.0016 (9)
C2	0.0230 (12)	0.0189 (9)	0.0154 (9)	−0.0001 (7)	0.0016 (8)	−0.0032 (7)
C3	0.0189 (12)	0.0286 (10)	0.0201 (11)	−0.0006 (9)	−0.0001 (9)	−0.0060 (9)
C4	0.0265 (13)	0.0236 (10)	0.0247 (11)	0.0069 (9)	0.0007 (10)	−0.0061 (9)
C5	0.0322 (15)	0.0238 (10)	0.0191 (10)	0.0031 (10)	0.0026 (11)	−0.0045 (8)
C6	0.0248 (12)	0.0252 (10)	0.0165 (10)	−0.0048 (9)	0.0005 (9)	−0.0045 (8)
C7	0.0231 (12)	0.0231 (9)	0.0122 (9)	−0.0011 (9)	0.0013 (9)	−0.0029 (8)
C8	0.0191 (11)	0.0221 (10)	0.0210 (10)	−0.0036 (8)	−0.0017 (9)	−0.0032 (8)
C9	0.0170 (11)	0.0199 (9)	0.0224 (10)	−0.0003 (8)	−0.0010 (9)	−0.0033 (8)
C10	0.0182 (11)	0.0150 (9)	0.0162 (10)	−0.0001 (8)	−0.0005 (9)	0.0006 (7)
C11	0.0197 (11)	0.0153 (8)	0.0126 (9)	−0.0008 (8)	0.0014 (9)	0.0012 (7)
C12	0.0161 (11)	0.0183 (9)	0.0188 (10)	0.0012 (8)	−0.0001 (9)	0.0013 (8)
C13	0.0142 (10)	0.0172 (8)	0.0172 (9)	0.0006 (7)	−0.0018 (9)	−0.0004 (9)
C14	0.0166 (9)	0.0138 (7)	0.0164 (8)	−0.0012 (9)	0.0004 (9)	−0.0009 (8)
C15	0.0213 (12)	0.0198 (9)	0.0180 (9)	−0.0014 (8)	0.0005 (9)	0.0044 (8)
C16	0.0142 (10)	0.0167 (9)	0.0167 (10)	−0.0002 (7)	0.0009 (8)	0.0015 (7)
C17	0.0174 (10)	0.0201 (9)	0.0211 (11)	0.0027 (8)	−0.0014 (8)	−0.0010 (8)
C18	0.0180 (11)	0.0178 (9)	0.0206 (10)	0.0013 (8)	0.0012 (8)	−0.0002 (8)
C19	0.0231 (12)	0.0226 (10)	0.0340 (12)	0.0070 (9)	−0.0008 (10)	−0.0039 (9)
C20	0.0207 (10)	0.0155 (8)	0.0155 (10)	0.0011 (7)	−0.0014 (8)	0.0015 (8)
C21	0.0198 (11)	0.0189 (8)	0.0195 (10)	0.0033 (7)	0.0026 (9)	−0.0041 (9)
C22	0.0254 (12)	0.0251 (10)	0.0207 (11)	−0.0049 (9)	−0.0005 (9)	−0.0005 (9)
C23	0.0232 (11)	0.0201 (9)	0.0235 (10)	0.0025 (7)	−0.0033 (10)	0.0004 (9)
C24	0.0259 (12)	0.0223 (9)	0.0393 (13)	−0.0027 (8)	−0.0008 (12)	−0.0035 (11)
C25	0.0328 (15)	0.0304 (11)	0.0450 (14)	−0.0057 (10)	0.0047 (12)	0.0026 (11)
C26	0.0166 (10)	0.0225 (9)	0.0191 (10)	−0.0013 (9)	−0.0005 (8)	0.0016 (9)
C27	0.0391 (15)	0.0229 (10)	0.0246 (11)	0.0033 (10)	0.0046 (10)	0.0065 (9)
C28	0.082 (2)	0.0397 (14)	0.0253 (12)	0.0159 (14)	0.0129 (15)	0.0137 (11)

### Geometric parameters (Å, °)

**Table:**

Br1—C11	2.0140 (17)	C12—H12A	1.0000
O1—C5	1.228 (2)	C13—C14	1.539 (2)
O2—C12	1.420 (2)	C13—H13A	0.9900
O2—H2	0.834 (9)	C13—H13B	0.9900
O3—C21	1.203 (2)	C14—C16	1.538 (3)
O4—C23	1.344 (3)	C14—C15	1.541 (2)
O4—C22	1.431 (2)	C14—C20	1.580 (2)
O5—C23	1.206 (2)	C15—H15A	0.9800
O6—C26	1.345 (2)	C15—H15B	0.9800
O6—C20	1.468 (2)	C15—H15C	0.9800
O7—C26	1.211 (2)	C16—C17	1.525 (3)
O1W—H11	0.842 (9)	C16—H16	1.0000
O1W—H12	0.842 (9)	C17—C18	1.543 (3)
C1—C2	1.567 (3)	C17—H17A	0.9900
C1—H1A	0.9800	C17—H17B	0.9900
C1—H1B	0.9800	C18—C19	1.526 (3)
C1—H1C	0.9800	C18—C20	1.571 (3)
C2—C3	1.490 (3)	C18—H18	1.0000
C2—C7	1.517 (3)	C19—H19A	0.9800
C2—C11	1.583 (2)	C19—H19B	0.9800
C3—C4	1.333 (3)	C19—H19C	0.9800
C3—H3	0.9500	C20—C21	1.529 (3)
C4—C5	1.459 (3)	C21—C22	1.521 (3)
C4—H4	0.9500	C22—H22A	0.9900
C5—C6	1.467 (3)	C22—H22B	0.9900
C6—C7	1.333 (3)	C23—C24	1.503 (3)
C6—H6	0.9500	C24—C25	1.504 (3)
C7—C8	1.498 (3)	C24—H24A	0.9900
C8—C9	1.537 (3)	C24—H24B	0.9900
C8—H8A	0.9900	C25—H25A	0.9800
C8—H8B	0.9900	C25—H25B	0.9800
C9—C10	1.529 (3)	C25—H25C	0.9800
C9—H9A	0.9900	C26—C27	1.496 (3)
C9—H9B	0.9900	C27—C28	1.493 (3)
C10—C16	1.529 (3)	C27—H27A	0.9900
C10—C11	1.541 (3)	C27—H27B	0.9900
C10—H10	1.0000	C28—H28A	0.9800
C11—C12	1.550 (3)	C28—H28B	0.9800
C12—C13	1.546 (3)	C28—H28C	0.9800
			
C12—O2—H2	107.7 (15)	C14—C15—H15B	109.5
C23—O4—C22	115.02 (15)	H15A—C15—H15B	109.5
C26—O6—C20	120.91 (14)	C14—C15—H15C	109.5
H11—O1W—H12	108.1 (14)	H15A—C15—H15C	109.5
C2—C1—H1A	109.5	H15B—C15—H15C	109.5
C2—C1—H1B	109.5	C17—C16—C10	119.09 (16)
H1A—C1—H1B	109.5	C17—C16—C14	103.05 (14)
C2—C1—H1C	109.5	C10—C16—C14	113.48 (15)
H1A—C1—H1C	109.5	C17—C16—H16	106.8
H1B—C1—H1C	109.5	C10—C16—H16	106.8
C3—C2—C7	112.70 (16)	C14—C16—H16	106.8
C3—C2—C1	105.68 (17)	C16—C17—C18	103.28 (15)
C7—C2—C1	106.38 (16)	C16—C17—H17A	111.1
C3—C2—C11	111.16 (16)	C18—C17—H17A	111.1
C7—C2—C11	107.50 (16)	C16—C17—H17B	111.1
C1—C2—C11	113.43 (14)	C18—C17—H17B	111.1
C4—C3—C2	124.9 (2)	H17A—C17—H17B	109.1
C4—C3—H3	117.6	C19—C18—C17	112.71 (16)
C2—C3—H3	117.6	C19—C18—C20	118.36 (16)
C3—C4—C5	120.9 (2)	C17—C18—C20	105.77 (15)
C3—C4—H4	119.5	C19—C18—H18	106.4
C5—C4—H4	119.5	C17—C18—H18	106.4
O1—C5—C4	122.3 (2)	C20—C18—H18	106.4
O1—C5—C6	121.3 (2)	C18—C19—H19A	109.5
C4—C5—C6	116.43 (17)	C18—C19—H19B	109.5
C7—C6—C5	123.2 (2)	H19A—C19—H19B	109.5
C7—C6—H6	118.4	C18—C19—H19C	109.5
C5—C6—H6	118.4	H19A—C19—H19C	109.5
C6—C7—C8	122.6 (2)	H19B—C19—H19C	109.5
C6—C7—C2	121.8 (2)	O6—C20—C21	109.42 (15)
C8—C7—C2	115.62 (16)	O6—C20—C18	108.99 (15)
C7—C8—C9	110.72 (17)	C21—C20—C18	117.93 (15)
C7—C8—H8A	109.5	O6—C20—C14	103.17 (13)
C9—C8—H8A	109.5	C21—C20—C14	111.98 (15)
C7—C8—H8B	109.5	C18—C20—C14	104.25 (15)
C9—C8—H8B	109.5	O3—C21—C22	120.75 (17)
H8A—C8—H8B	108.1	O3—C21—C20	121.59 (18)
C10—C9—C8	112.12 (17)	C22—C21—C20	117.53 (16)
C10—C9—H9A	109.2	O4—C22—C21	111.44 (16)
C8—C9—H9A	109.2	O4—C22—H22A	109.3
C10—C9—H9B	109.2	C21—C22—H22A	109.3
C8—C9—H9B	109.2	O4—C22—H22B	109.3
H9A—C9—H9B	107.9	C21—C22—H22B	109.3
C9—C10—C16	110.74 (16)	H22A—C22—H22B	108.0
C9—C10—C11	112.43 (15)	O5—C23—O4	122.26 (18)
C16—C10—C11	110.33 (16)	O5—C23—C24	125.7 (2)
C9—C10—H10	107.7	O4—C23—C24	112.03 (17)
C16—C10—H10	107.7	C23—C24—C25	113.52 (17)
C11—C10—H10	107.7	C23—C24—H24A	108.9
C10—C11—C12	113.44 (14)	C25—C24—H24A	108.9
C10—C11—C2	111.36 (15)	C23—C24—H24B	108.9
C12—C11—C2	115.16 (16)	C25—C24—H24B	108.9
C10—C11—Br1	108.15 (13)	H24A—C24—H24B	107.7
C12—C11—Br1	102.88 (12)	C24—C25—H25A	109.5
C2—C11—Br1	104.87 (11)	C24—C25—H25B	109.5
O2—C12—C13	112.67 (15)	H25A—C25—H25B	109.5
O2—C12—C11	107.10 (15)	C24—C25—H25C	109.5
C13—C12—C11	113.39 (16)	H25A—C25—H25C	109.5
O2—C12—H12A	107.8	H25B—C25—H25C	109.5
C13—C12—H12A	107.8	O7—C26—O6	122.58 (18)
C11—C12—H12A	107.8	O7—C26—C27	126.86 (18)
C14—C13—C12	112.99 (16)	O6—C26—C27	110.56 (17)
C14—C13—H13A	109.0	C28—C27—C26	114.81 (19)
C12—C13—H13A	109.0	C28—C27—H27A	108.6
C14—C13—H13B	109.0	C26—C27—H27A	108.6
C12—C13—H13B	109.0	C28—C27—H27B	108.6
H13A—C13—H13B	107.8	C26—C27—H27B	108.6
C16—C14—C13	108.75 (13)	H27A—C27—H27B	107.5
C16—C14—C15	112.57 (15)	C27—C28—H28A	109.5
C13—C14—C15	111.15 (16)	C27—C28—H28B	109.5
C16—C14—C20	99.10 (14)	H28A—C28—H28B	109.5
C13—C14—C20	115.80 (15)	C27—C28—H28C	109.5
C15—C14—C20	108.99 (13)	H28A—C28—H28C	109.5
C14—C15—H15A	109.5	H28B—C28—H28C	109.5
			
C7—C2—C3—C4	0.7 (3)	C11—C10—C16—C17	178.77 (15)
C1—C2—C3—C4	−115.0 (2)	C9—C10—C16—C14	−177.67 (15)
C11—C2—C3—C4	121.5 (2)	C11—C10—C16—C14	57.2 (2)
C2—C3—C4—C5	−2.9 (3)	C13—C14—C16—C17	170.68 (15)
C3—C4—C5—O1	−176.2 (2)	C15—C14—C16—C17	−65.69 (18)
C3—C4—C5—C6	4.1 (3)	C20—C14—C16—C17	49.36 (16)
O1—C5—C6—C7	176.8 (2)	C13—C14—C16—C10	−59.2 (2)
C4—C5—C6—C7	−3.5 (3)	C15—C14—C16—C10	64.46 (19)
C5—C6—C7—C8	−178.16 (18)	C20—C14—C16—C10	179.52 (15)
C5—C6—C7—C2	1.6 (3)	C10—C16—C17—C18	−170.04 (16)
C3—C2—C7—C6	0.0 (3)	C14—C16—C17—C18	−43.38 (18)
C1—C2—C7—C6	115.3 (2)	C16—C17—C18—C19	149.83 (17)
C11—C2—C7—C6	−122.9 (2)	C16—C17—C18—C20	19.04 (19)
C3—C2—C7—C8	179.71 (17)	C26—O6—C20—C21	69.4 (2)
C1—C2—C7—C8	−64.9 (2)	C26—O6—C20—C18	−60.9 (2)
C11—C2—C7—C8	56.9 (2)	C26—O6—C20—C14	−171.26 (16)
C6—C7—C8—C9	123.4 (2)	C19—C18—C20—O6	134.07 (17)
C2—C7—C8—C9	−56.3 (2)	C17—C18—C20—O6	−98.47 (16)
C7—C8—C9—C10	51.6 (2)	C19—C18—C20—C21	8.6 (3)
C8—C9—C10—C16	−176.37 (16)	C17—C18—C20—C21	136.05 (17)
C8—C9—C10—C11	−52.4 (2)	C19—C18—C20—C14	−116.29 (18)
C9—C10—C11—C12	−173.84 (16)	C17—C18—C20—C14	11.17 (18)
C16—C10—C11—C12	−49.7 (2)	C16—C14—C20—O6	77.41 (15)
C9—C10—C11—C2	54.3 (2)	C13—C14—C20—O6	−38.62 (19)
C16—C10—C11—C2	178.48 (14)	C15—C14—C20—O6	−164.80 (14)
C9—C10—C11—Br1	−60.40 (18)	C16—C14—C20—C21	−165.02 (15)
C16—C10—C11—Br1	63.76 (16)	C13—C14—C20—C21	78.94 (19)
C3—C2—C11—C10	−177.98 (16)	C15—C14—C20—C21	−47.2 (2)
C7—C2—C11—C10	−54.21 (19)	C16—C14—C20—C18	−36.43 (16)
C1—C2—C11—C10	63.1 (2)	C13—C14—C20—C18	−152.47 (14)
C3—C2—C11—C12	51.1 (2)	C15—C14—C20—C18	81.36 (18)
C7—C2—C11—C12	174.83 (15)	O6—C20—C21—O3	−168.22 (17)
C1—C2—C11—C12	−67.9 (2)	C18—C20—C21—O3	−42.9 (3)
C3—C2—C11—Br1	−61.25 (18)	C14—C20—C21—O3	78.0 (2)
C7—C2—C11—Br1	62.53 (16)	O6—C20—C21—C22	15.8 (2)
C1—C2—C11—Br1	179.84 (14)	C18—C20—C21—C22	141.12 (18)
C10—C11—C12—O2	−78.15 (19)	C14—C20—C21—C22	−97.9 (2)
C2—C11—C12—O2	51.8 (2)	C23—O4—C22—C21	−75.7 (2)
Br1—C11—C12—O2	165.26 (11)	O3—C21—C22—O4	−13.0 (3)
C10—C11—C12—C13	46.8 (2)	C20—C21—C22—O4	162.93 (17)
C2—C11—C12—C13	176.72 (15)	C22—O4—C23—O5	0.3 (3)
Br1—C11—C12—C13	−69.82 (16)	C22—O4—C23—C24	−179.85 (17)
O2—C12—C13—C14	72.5 (2)	O5—C23—C24—C25	−26.0 (3)
C11—C12—C13—C14	−49.3 (2)	O4—C23—C24—C25	154.08 (19)
C12—C13—C14—C16	54.1 (2)	C20—O6—C26—O7	−9.0 (3)
C12—C13—C14—C15	−70.40 (19)	C20—O6—C26—C27	171.58 (17)
C12—C13—C14—C20	164.53 (15)	O7—C26—C27—C28	−3.2 (4)
C9—C10—C16—C17	−56.1 (2)	O6—C26—C27—C28	176.3 (2)

### Hydrogen-bond geometry (Å, °)

**Table:**

*D*—H···*A*	*D*—H	H···*A*	*D*···*A*	*D*—H···*A*
O2—H2···O1w	0.83 (1)	1.93 (1)	2.759 (2)	173 (2)
O1w—H11···O5	0.84 (1)	2.06 (1)	2.859 (2)	159 (3)
O1w—H12···O7^i^	0.84 (1)	2.03 (1)	2.854 (2)	168 (2)

Symmetry codes: (i) −*x*+3/2, −*y*+2, *z*−1/2.
